# Eliminating Trachoma in Africa: The Importance of Environmental Interventions

**DOI:** 10.7759/cureus.52358

**Published:** 2024-01-16

**Authors:** Ahmed Ageed, Maaz Khan

**Affiliations:** 1 Hospital Medicine, University Hospitals of Leicester NHS Trust, Leicester, GBR; 2 Medical Education, Royal Surrey County Hospital, Guildford, GBR

**Keywords:** vision 2020, trichomonas vaginalis, hygeine, environmental sanitation, water, trachoma

## Abstract

Subsequent to the failure of the World Health Organisation (WHO) of achieving their target to eliminate trachoma by the year 2020, the most effective strategy in eliminating trachoma must be re-examined to accomplish the new target of eradication by the year 2030. Whilst antibiotic therapy is a core foundation of this elimination strategy, another important factor is the state of the environmental conditions in trachoma endemic countries. This manuscript aimed to identify the impact of environmental improvement strategies on the prevalence of trachoma and the significance of environmental improvement alongside the use of antibiotic treatment to achieve trachoma elimination. Two independent literature searches were conducted up until the 5th of July 2021. Two main databases were used to carry out these literature searches, namely, Ovid EMBASE and Ovid MEDLINE. All of the relevant references were found using MeSH and free text terms. Key terms used were 'trachoma', 'water', 'sanitation', 'hygiene' and 'environmental Improvement'. The exclusion criteria included non-African-based studies, review papers, protocols and case reports. A total of 17 studies were included for this review. Living within a close range of a water source was significantly associated with reduced risk of trachoma infection. Water obtained from piped water sources was associated with the lowest rates of active trachoma. Studies on facial cleanliness evidenced a strong association with reduced prevalence of trachoma. Whilst the provision of latrine facilities found was significantly associated with reduced prevalence of trachoma, there was no significant difference between the use of private latrine facilities over communal latrine facilities. The use of repeated scheduled antibiotic treatments over single-use antibiotic distribution had a greater impact both short term and long term on the prevalence rates of trachoma. Nonetheless, prevalence rates increased again following the commencement of treatment. Mass antibiotic treatment has been proven to have a greater impact on lowering the prevalence of trachoma initially, but this impact is not sustainable due to the rise in prevalence rates following the completion of treatment. A holistic approach, therefore, must be implemented with evidence showing that an emphasis on longer-term environmental methods should be implemented to compliment antibiotic distribution. Prioritisation of specific interventional measures should be tailored according to local epidemiology; nonetheless, these measures form the backbone of a trachoma elimination strategy to eliminate trachoma by the year 2030.

## Introduction and background

Trachoma is the largest cause of infectious blindness in the world and is a part of a group of diseases known as neglected tropical diseases (NTDs) [1]. According to the World Health Organisation (WHO), trachoma remains a public health crisis in 44 countries worldwide [2,3] and is responsible for the blindness or visual impairment of approximately 1.9 million people [4]. Trachoma, most commonly present in areas of strife poverty, is a disease that manifests itself in areas of poor-quality water supply, hygiene and sanitation facilities, making the rural and most isolated communities of sub-Saharan Africa particularly vulnerable. According to some of the latest figures, approximately 170 million of the African population are at risk of developing trachoma, with 72% of the world’s most severe, blinding and end-stage cases of trachoma found in the continent [5]. 

Trachoma, caused by repeated infection of the serological variants A-C of *Chlamydia trachomatis*, is a form of chronic conjunctivitis that ordinarily presents bilaterally and is characterised by progressive exacerbations and remissions [6]. During the initial phase, the ‘active inflammatory phase’, trachoma presents with follicular conjunctivitis mostly prominent in the superior tarsal plate alongside pannus formation. The active phase of trachoma can cause conjunctival scarring and ultimately lead to the ‘chronic cicatricial phase’ consisting of trachomatous trichiasis and corneal opacities, both of which can cause irreversible visual impairment or blindness. 

No longer present in the world’s most developed countries, the burden of this disease has fallen upon nations that are the most poorly equipped to tackle its threat to their populations. The tremendous economic strain and failure to provide much of the African population with basic water and sanitation facilities has created a breeding ground for the transmission of trachoma via its two main pathways: direct contact and flies [7]. Eye, nose and throat secretions are the main forms of direct contact of which trachoma can be spread; however, direct contact can also occur in the form of shared towels, handkerchiefs or clothes. The *Musca sorbens* species of flies are known to act as a vector for trachoma by carrying the causative bacterium *Chlamydia trachomatis*. These flies are known as ‘eye-seeking’ as they feed on individuals' ocular and nasal discharge in order to obtain their nutrients [8].

The spread of trachoma can therefore be limited by controlling two types of factors: personal and environmental. It is of vital importance that the population in endemic countries are educated on personal measures to limit trachoma transmission, such as hand and facial cleanliness. Nonetheless, whilst efforts to improve personal hygiene are important, their effects are very much limited by the environmental resources available to those in trachoma-endemic regions. With flies being the main vector for trachoma transmission, there is a direct correlation between maintaining environmental cleanliness and trachoma transmission as ‘eye-seeking’ flies are attracted to areas of poor hygiene.

Medical and surgical intervention for trachoma is readily available and is included in the WHO trachoma treatment acronym ‘SAFE’ to eliminate trachoma by 2020. The acronym stands for 'Surgery (for advanced disease), Antibiotics, Facial cleanliness and Environmental improvement'. The wide distribution of antibiotics to all susceptible is recommended to limit trachoma transmission, including those already affected. Whilst antibiotic treatment is effective, it does not provide a long-lasting cure, as those treated by antibiotics can become reinfected again in the future, which can then still inevitably cause irreversible visual impairment or blindness. The surgical procedure for trachoma is called a bilamellar tarsal rotation procedure (BTRP). This surgery involves the making of a full-thickness incision and rotating the distal eyelid fragment by suture placement. The aim of this surgery is to correct any trichiasis (inward eyelash growth) secondary to trachoma [9]. Unfortunately, endemic countries do not have the economic power to fund the widespread facilitation of such surgical procedures, and more importantly, those who require surgery tend to have reached a severe point of disease progression, which ideally should not have been reached in the first place. 

This consequently takes us back to the fundamental principle of modern healthcare, 'prevention is better than cure'. Whilst the S and A components of the SAFE acronym focus on curative measures, the F and E components focus on preventative measures and therefore should be regarded as the main focus in the fight to eliminate trachoma. It is for this reason that the WHO set out to improve three environmental factors with the aim of eliminating trachoma; these factors are improving access to safe Water, Sanitation, and Hygiene (WASH) [10].

The importance of access to clean water cannot be understated in the fight against trachoma. Water not only acts as a drinking source but also as a sanitation source for the population facing an environment needing to prevent the attraction of flies that transmit this disease in particular. With trachoma generally presenting in the drier parts of the world, providing wide availability of good quality water supplies is a difficult challenge. For this reason, establishing solid evidence that appropriate water supply improvement can help reduce the rates of trachoma is of paramount importance in order to justify fighting such an uphill battle. The relationship between water supplies and trachoma can often be more complex than it may initially appear. This dissertation will explore trachoma, its relationship with water and other interventional measures to determine the best approach to eliminating this disease from an efficacy, longevity and cost-effectiveness point of view.

Clinical features of trachoma 

The clinical characteristics of trachoma are identified in five stages of development as outlined by the WHO: trachomatous inflammation follicular (TF), trachomatous inflammation intense (TI), trachomatous scarring (TS), trachomatous trichiasis (TT) and corneal opacity (CO) [11].

The first stage, TF, is defined as 'the presence of five or more follicles in the upper tarsal conjunctiva (UTC)'. These follicles may be white, grey or yellow elevations with the follicles themselves appearing paler than the surrounding conjunctiva [12].

The progression from TF to TI is characterised by the thickened, red and rough appearance of the UTC. At this stage, the blood vessels normally visible in the UTC can no longer be seen and are, instead, hidden by a diffuse appearance of inflammatory follicles [11]. 

These follicles eventually disappear and leave behind scars in the stage known as TS. These conjunctival scars can alternatively be referred to as ‘Arlt’s line’, named after the famous Austrian ophthalmologist Carl Ferdinand von Arlt. Arlt’s line appears horizontally at the junction of the anterior one-third and posterior two-thirds of the conjunctiva and runs parallel to the eyelid [13].

The conjunctival scarring from TS then causes entropion (inversion of the eyelid). This causes the eyelashes to rub against the cornea leading to ulcerations and chronic inflammation. This stage is known as TT [11]. Eventually, due to the damage caused by TT, the cornea slowly becomes cloudy which causes irreversible visual impairment and even blindness [11].

Epidemiology 

It is estimated that trachoma affects at least 1.9 million people worldwide across 44 different countries and is regarded by the WHO as the leading global cause of infectious blindness [3,4]. A recent WHO epidemiological report approximated that 136.9 million people are inhabited in endemic regions for trachoma. Despite such an alarming figure, the WHO reports that the number of people at risk from trachoma decreased by 5.3 million between 2019 and 2020 [13]. The prevalence of persons with TT, the late blinding phase of trachoma, has been recorded to be two million as of May 2020 [13].

Trachoma was eliminated from North America and Western Europe in the 19th century as a result of improved living standards, yet it still remains a huge burden on less economically developed countries. Geographically, the greatest active trachoma rates (TF and/or TI) exist in sub-Saharan Africa, making up 72% of worldwide cases [5]. Trachoma is believed to be endemic within the regional area of the Sahel belt, which crosses from the west to the east regions of sub-Saharan Africa [14]. It is reported that Ethiopia and Sudan are two of the countries with the upper most percentage of cases for active trachoma, present in over 50% of children under the age of 10 [15,16]. Despite the already alarming figures, these numbers are predicted to actually be much higher as large areas suspected to be endemic are unmapped in these countries.

The presence of trachoma as a public health problem reaches much further than Africa alone, with many countries across Asia and Central and South America also being affected. The WHO estimates that approximately 49 million children in the Asia-Pacific region have active trachoma, with over 3.5 million adults having trichiasis [17]. On a gross scale, it is easy to state that the presence of trachoma is simply an economical issue due to the wealth disparity between the developed and developing worlds. However, within the continent of Africa itself, it has been found that there is not a linear pattern between richer nations and lower prevalence of trachoma. Only two countries have eliminated trachoma in sub-Saharan Africa, Ghana and the Gambia, yet according to the latest statistical analysis in the gross domestic product (GDP) per capita, these countries fall in the 17th and 39th positions, respectively. This emphasises the importance of strategic resource distribution of which richer African countries have failed to implement, thus leading to a failure in the eradication of trachoma in these nations. 

Age and gender 

A study into trachoma risk factors conducted in the hyperendemic regions of Guinea-Bissau found that children under the age of 10 are at a far higher probability of active trachoma infection than those elder to them [18]. Of 293 households, 1,511 participants were randomly selected across 39 villages, and children under the age of 10 made up 40.9% of the total number of active trachoma cases. Within this younger age group, however, there were further differences found between children aged zero to five years and children aged six to 10 years. The number of *Chlamydial trachomatis* ocular infection with cases in the zero to five years' age group was 66% greater (16) in comparison to the six to 10 years' age group (250) [18]. These findings are consistent with the other available literature, which also supports the notion that children of younger ages are the main pool of this disease [19].

The suspected procurement of immunity in childhood can explain the discrepancy found in the link between conjunctival inflammation and infection. This childhood immunity gained may then lead to reducing the incidence rates of clinically active trachoma although there is still a strong association with the infection. Another likely contributing factor is that generally older children are more aware about the hygienic measures required to prevent trachoma infection than younger children.

Almost all survey data from trachoma-endemic countries are in a unanimous agreement that trachoma-related blindness presents higher in women compared to men. One study conducted in Ethiopia found that women made up 75% of cases for trichiasis within the community and had an excess risk of corneal opacification and scarring [20]. Whilst the evidence shows a strikingly apparent difference previously between men and women, the difference is a lot more subtle when comparing between younger boys and younger girls. One study showed that whilst 11 out of 18 trichiasis cases were women, amongst children under the age of 10, 21.1% of boys and 13.5% of girls had active trachoma [21]. An additional study revealed that pre-school girls were only slightly more at risk of active trachoma than their same-age male counterparts, whereas female adults had a double excess risk for inflammatory trachoma in comparison to male adults [22].

This age-related gender gap in the prevalence of active trachoma has been attributed to a multitude of reasons found in available literature. Research has not shown any female biological susceptibility to trachoma over males; be that as it may, what is known is that women are more predisposed to developing dry eye syndrome (DES), due to hormonal imbalances, especially in the case of women of menopausal age [23]. It has therefore been theorised that because of this susceptibility to DES, women with trichiasis are more likely to have corneal damage than men. Due to the lack of data analysed by sex, the ability to confirm this theory remains unachievable.

The effect of gender roles on the increased risk of active trachoma in women has also been demonstrated to be a likely risk factor. In the majority of endemic countries, the cultural norm is for the women to be the primary caregivers of the household, spending a lot of her time with the children, whereas the male is much more likely to be the working breadwinner, thus spending less time exposed to the children. Given that children are the most susceptible group to active trachoma [19], this would also mean that they are also the group most likely to transmit the disease, leaving the caregiving women particularly at risk.

Education**

Whilst women and particularly mothers have been shown to be vulnerable to *Chlamydia trachomatis* infection, they are also in a leading position to tackle its transmission. Educated mothers, who are aware of the importance of sanitation and cleanliness, have been found to have a significantly lower prevalence of trachoma vector parasites within their family parameters in comparison to mothers that were uneducated [24]. 

Healthcare education has been promoted heavily by environmental intervention campaigns in the fight to eradicate trachoma [25]. Whilst the prevalence of trachoma is heavily influenced by factors out of control for those living in poverty, there are controllable measures that can be implemented to reduce the risk of both infection and transmission. This includes the components ‘F’ and ‘E’ of the SAFE treatment acronym that was initiated by the WHO to tackle trachoma. The success of the ‘F’ and ‘E’ components, standing for ‘facial cleanliness’ and ‘environmental improvement’, are heavily dependent on public engagement, and therefore education campaigns have been on the forefront of the WHO’s strategy to combat trachoma.

One of the main practical elements of these campaigns was to implement healthcare education via the school curriculum. Schools have repeatedly been identified as an entry point to access a large proportion of the general population directly through schoolchildren and then indirectly to their families and other community members. The available literature on the influence of school health education on trachoma has shown to be effective, with students enthusiastic to implement new behaviours into practice [26].

One study conducted within 40 rural communities in rural Ethiopia looked at the effect of a health education programme on the rates of inflammatory trachoma in children aged three to nine years [27]. Non-governmental organisation (NGO) activities based on implementing the SAFE strategy were conducted in 30 of the 40 communities, with all 40 receiving health education via radio broadcast and 10 communities receiving enhanced educational messages using videos. Overall, there was a small but statistically substantial (P<0.001) 8% reduction in the prevalence of active trachoma after education intervention measures were implemented. This truly highlights the importance of hygiene education within the education system [27].

Global elimination of trachoma by 2020 

Launched in 1999, a global initiative formed by both the WHO and the International Agency for the Prevention of Blindness (IAPB) was established to eliminate all avoidable blindness by the year 2020 called ‘Vision 2020: The Right to Sight'. This global campaign targeted the world’s leading causes of blindness including trachoma, as well as onchocerciasis, congenital blindness, refractive error and low vision [28]. Vision 2020 was built upon the groundwork of societal engagement and use of national programmes. The main focus of these programmes are to implement and utilise practical infection management interventions, anthropological resource development and infrastructure advancement [29].

In the specific case of trachoma, the WHO established the Global Elimination of Trachoma by the year 2020 (GET 2020), which was an international alliance of interested parties, governments and international governmental organisations and NGOs. GET 2020 is a partnership with the aim of supporting countries’ application of the SAFE treatment strategy. Particular emphasis was placed upon environmental improvement including, but not limited to, improving water, sanitation and hygiene (WASH) conditions in trachoma-endemic countries. This dissertation will discuss the aspects of environmental improvement tackled by GET 2020, its success in lowering the prevalence of trachoma and possible feasible actions for the future.

Trachoma and water

The correlation between trachoma and water is more complicated than it may initially appear and can manifest itself in many ways. The transmission of trachoma most commonly occurs in arid areas with poor access to water [30]. There are many reasons why improvements in water supply in these areas would lead to a reduction in the trachoma prevalence within a community from the use of water as a cleaning source to the control fly infestation.

Trachoma is transmitted by a species of flies known as the ‘*M. sorbens*’ fly. The current evidence suggests that the flies are attracted to ocular and nasal discharge as they are attracted to sources of moisture [31]. By improving dry conditions, including water thrown on the ground, an alternative source of moisture will be provided for flies, which otherwise would seek it on the ocular and nasal discharge of children [10].

The facial cleanliness aspect of the SAFE treatment strategy can only be achieved with access to increased clean water availability. With children’s faces being the most common sites of re-infection of *Chlamydia trachomatis*, clean water being made available means that faces can be cleaned more thoroughly and frequently [32].

The impact of facial cleanliness on clinical trachoma is demonstrated in a study conducted in two villages in a hyper-endemic region of Central Tanzania [33]. A total of 472 children of pre-school age were assessed for particular signs of unclean faces and the presence of trachoma. These signs included nasal discharge and the presence of flies; other signs were said to be of no importance. The findings showed that the odds of trachoma in a family increased by 42% if a sibling had trachoma and a face is unclean, compared to if the sibling just had trachoma alone, highlighting the importance of a need for access to clean water as a means for facial cleanliness [33]. 

Not only is access to water important but also the source from which it is accessed from. Strong independent associations are shown between active trachoma and types of household water source. One study conducted in Senegal showed that children living in compounds with access to a public outdoor tap as their principal supply of water were more probable to have unclean faces than children with private indoor taps [34]. Another factor of private versus public sources of water is the amount of water resources available to a single household. A study conducted in Guinea-Bissau showed that household access only to a single water source or natural spring only (compared to households with various water sources) was strongly connected with *C. trachomatis* infectivity [18].

Walking distance to the nearest water source has been investigated for an impact on the prevalence of trachoma. A study in Ethiopia looked into this exact matter and found that contrary to what might be predicted, those living further than 15 minutes walking distance from a source of water had a lesser amount of inflammatory trachoma than those with a water supply nearer to home [35]. The negative study results could be explained by the reason that improvements in hygiene do not automatically follow after the supply of a conveniently located water source and thus emphasises the importance of other hygiene aspects, such as sanitation facilities and fly population control. 

Sanitation and hygiene 

According to a 2017 report by ‘WaterAid’, all 10 of the world’s worst countries for access to basic facilities for the safe human waste removal (faeces and urine) are in sub-Saharan Africa, where only 28% of individuals have access to adequate sanitation facilities [36]. The fact that sub-Saharan Africa is also the most endemic region for trachoma does not necessitate that there is an association between trachoma and poor sanitation facilities; however, according to several studies, this does appear to be the case. Studies have assessed the effectiveness of environmental sanitary measures on the prevalence of trachoma in endemic areas and found that endemic regions with a higher proportion of community latrine usage were most likely to experience a reduction in the prevalence of ocular chlamydia [37-39].

Unhygienically maintained sanitation facilities are sites of attraction for eye seeking flies. These flies act as mechanical vectors for *C. trachomatis *by picking up pathogens from infectious material and transferring them to an uninfected host. Studies that have looked into the effectiveness of insecticide spray as a fly population control measure have uncovered an association with a reduced prevalence of inflammatory trachoma [39].

Nonetheless, similar to the same way that not all mosquitoes transmit malaria, not all flies transmit *C. trachomatis*. Evidence suggests that the most likely species of fly responsible for the transmission of trachoma is the *M. sorbens* species [40]. One study found that the prevalence of trachoma fell significantly when *M. sorbens* was removed from the environment where this species of fly is usually present all year round [31]. This therefore means, however, that the success of fly population control can vary from region to region dependent on the species of flies that naturally inhabit within a region as the removal of other species of flies will not impact the prevalence of trachoma [31]. 

The use of insecticide spray is not the only method to control fly population, and studies have found an association between appropriate latrine access and reduced number of flies. A study conducted in Egypt discovered that less trachoma was found in households in which simple pit latrines were present [41]. The reason behind this is said to be that *M. sorbens* breeds in solid excrements on the ground, and therefore the provision of latrines where contents liquefy rapidly solves this issue. However, with the estimations that 215 million sub-Saharan Africans continue to openly defecate, providing latrine access could play a major role in the combat against trachoma [42].

## Review

Methodology

**Figure 1:**
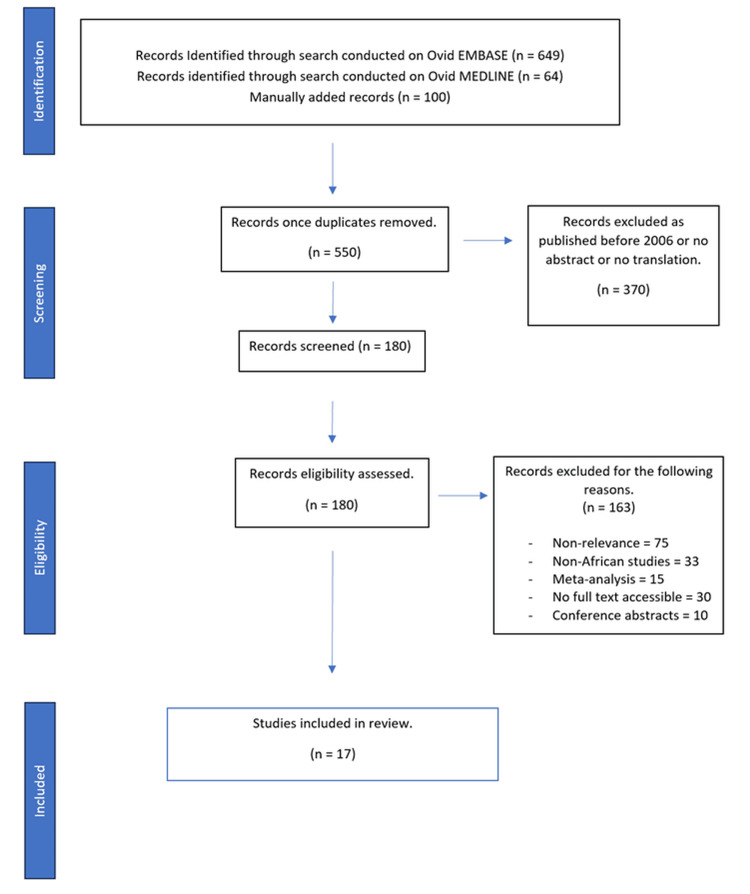
PRISMA flow chart diagram PRISMA: Preferred Reporting Items for Systematic Reviews and Meta-Analyses

A literature search was conducted up until the 5th of July 2021. The two main databases that the literature search was carried out on were Ovid MEDLINE (see Appendix A) and Ovid EMBASE (see Appendix B). The key concepts used to search were 'trachoma', 'environmental improvement', 'water', 'sanitation' and 'hygiene'. MeSH and free text terms were used in order to collate all the relevant references. The references were then exported to an end note in order to remove duplicates, leaving 550 references to be screened. Studies were then assessed in terms of eligibility; the eligibility criteria ensured that only articles in English, based in Africa and published in the last 15 years (2006-2021) were selected. The articles must also meet the criteria that they are not review papers and conference abstracts. The papers must also focus on trachoma only and not neglected tropical ocular diseases in general and must focus on environmental intervention as opposed to surgical or medical methods. The studies must have a minimum of 12 months of follow-up to show the effectiveness of the intervention. The steps are summarised in the Preferred Reporting Items for Systematic Reviews and Meta-Analyses (PRISMA) flow chart (Figure 1) [43]. After exclusion, the total number of studies that were selected is 17.

Results

Distance to Water Source

Baggaley et al. conducted a study investigating the relationship between the incidence of inflammatory trachoma in children in the Rambo district of Tanzania and proximity to the closest source of water [44]. From the 64 villages in Rambo, 10 groups of between eight and 40 households large were randomly selected, and examinations were conducted looking for physical signs of active trachoma in all children aged one to nine. In total, 12,415 children were examined, and the results found 1,171 cases of active trachoma. From these 1,171 cases, it was found that a greater distance to the nearest water source was significantly associated with a rise in the prevalence of trachoma (p-value =<0.0001) even after the accountability for confounding variables, such as age, gender and ethnicity [44].

A similar study conducted by West et al. investigated the point at which distance became a significant factor in the prevalence of trachoma. An epidemiological survey was conducted into the risk factors for trachoma in Tanzania across 20 villages. Risk factors evaluated included distance to water source and other factors, such as quality of water and types of water sources. These factors were then assessed for an association with the cleanliness of children’s faces and prevalence against trachoma. The study assessed distance to water source via three different time categories, <30 minutes, 0.5-2 hours and >2 hours. The results showed that the ratio of households where all children were had trachoma risen with the time required to collect water from 37% amongst participants who lived <30 minutes, to 49% for those that were inhabited 0.5-2 hours away, to 50% amongst participants that lived >2 hours away from the nearest water source. Moreover, the proportion of households where no children were infected with trachoma decreased as the distance to the nearest source of water increased [45]. 

Type of Water Source

The cleanliness of water collected has closely been associated with the type of water source that the water has been acquired from. A study by Niguise et al. that took place in the Gonji Kolella region of North West Ethiopia investigated the association between the type of water source and the prevalence of active trachoma. The total number of children aged one to nine included in this was 618. Data were gathered using presented and structured questionnaires and observation. The common sources of water amongst the population that were in this study were water from pipes (70.2%), protected wells (18.6%), protected springs (6.3%) and unprotected springs (4.9%). The results directly compared water from a pipe against the other three sources and found that water that was not derived from a piped water source was significantly related with active trachoma (P < 0.001) [46].

Similar findings were seen in the study conducted by Golovaty et al. [47]. This study assessed the active trachoma prevalence amongst 507 children from 232 households between the ages of one to nine years in Ankober, Ethiopia, and looked for associations between different types of water sources. Again, this study compared piped water to other water sources, including spring water and water from rivers or lakes. The results showed that active trachoma was present in 18.5% of children that had access to piped water compared to 28% with access to spring water and 53.5% with access to river or lake water. A significant association was found between active trachoma and a lack of access to piped water. Overall, both studies show evidence that piped water is associated with the lowest rates of active trachoma in comparison to other sources. 

Facial Cleanliness and Fly Infestation 

A study conducted in Nigeria randomly selected 12 villages, and a census of all children in households aged between one and five years were selected at random and assessed for several trachoma and ocular *Chlamydia trachomatis* (*C. trachomatis*) infection risk factors [48]. A criteria for a ‘clean’ face was the 'absence of ocular and nasal secretions and food on the face'. The presence of flies did not classify the patient's face as unclean, but the results were reported in three categories: clean, clean but with flies and unclean. The percentages of children aged one to five with trachoma was 22.1% for children with clean faces, 41.9% for children with clean faces but with flies and 48.3% for children with unclean faces. The percentage of children aged one to five with *C. trachomatis* infection with a clean face was 5.2%, that of children with a clean face but with flies was 16.5% and that of children with an unclean face was 27.1%. Children with clean faces but with flies on their face had a greater chance of having trachoma in comparison to children with clean faces without the presence of flies, yet there was no association of facial flies with infection (P-value = 0.23) [48].

Another study conducted in Kongwa, Tanzania, explored the impact of face washing intervention programmes on the prevalence of trachoma in children aged one to seven. Three pairs of villages were randomised - one of each pair would receive the face washing intervention programme alongside antibiotic treatment, whereas the other village would receive antibiotic treatment alone. The programme was created to increase the practice of face washing amongst the village youth. Meetings were organised within the neighbourhood to educate adults on the importance of face washing for children as an imperative trachoma control measure. Other intervention activities, including school assemblies and classes with the traditional therapists, were organised in the campaign for facial cleanliness. These intervention activities were conducted for the duration of one month both during and after antibiotic treatment. A total of 1,417 children participated in this study, and the results showed that there was an average 10% increase across the three pairs of villages in children with sustained clean faces. The active and severe trachoma prevalence rates at one year were then analysed across the three villages to assess whether the face washing intervention programmes were successful in lowering trachoma prevalence rates. The odds of trachoma at one year for the children in the intervention group was 0.62 [49]. 

The effectiveness of different face washing techniques on the removal of ocular and nasal discharge has also been investigated by a study conducted in Ethiopia in 2018 [50]. Children with TF/TI aged between one and nine years were included in this research. A total of 247 households where there was a minimum of one child between the ages of one and nine years with TF or TI were randomly selected. In total, 83 children were enrolled and assigned to any of four unique face cleaning procedures. The different procedures were as follows: 1) face washing with water only ('washed with water'), 2) face washing with soap and water ('washed with soap'), 3) face wiped by the caregivers’ hand ('wiped with hand') followed by handwashing for up to 30 seconds with water or 4) face washing with water and soap [50].

Latrine Use 

A study conducted in Darfur, Sudan, in 2019 evaluated the association between the presence of TF and TI in children and several risk factors [51]. The impact of open defecation on trachoma was evaluated amongst other independent risk factors including but not limited to water access and household crowding. A secondary evaluation of data from 27 trachoma prevalence surveys was conducted during 2014-2015 in the Darfur states. Household-level data on facilities for sanitation were gathered by field teams via an interview with the head of household. TF was found to be independently associated with the practice of open defecation (odds ratio (OR) 3.1, 95% confidence interval (CI) 1.1 to 8.6) [51].

The association between open defecation and prevalence of trachoma has led to intervention measures for the provision of latrine facilities to be put in place to assess whether this could improve the prevalence rates of trachoma. In Amhara, Ethiopia, between 2011 and 2014 a study was performed that investigated the relationship between the active trachoma prevalence in children between the ages of one and nine years and the use of community sanitation facilities. Data were collected on observed displays of latrine usage into a measure of community sanitation usage calculated as the proportion of households with a latrine in use. Clinical signs of active trachoma were looked for in all household members, such as TI and TF, as an outcome variable for trachoma.

The results were split by percentage of households within a cluster (approximately 40 households) that showed evidence of sanitation use. The results were documented in five different categories: <20% usage, 20-40% usage, 40-60% usage, 60-80% usage and >80% usage. Community sanitation usage in the 60-80% and >80% groups were individually associated with lower prevalence odds for active trachoma in comparison to the <20% usage group (OR: 0.76; 95% CI: 0.57-1.03 and OR: 0.67; 95% CI: 0.48-0.95, respectively). The study concluded that the greater the presence of community sanitation use, the lower the prevalence rates of active trachoma amongst children [37].

Community latrine sharing is a common practice in sub-Saharan Africa, but this has led to some questioning as to whether the provision of private latrines as opposed to community latrines would be a better approach to eliminating trachoma, despite being more expensive to fund. A study conducted in rural Tanzania in 2007 investigated whether there is any disparity between the use of shared versus private latrines in the decreasing of trachoma risk. The study was conducted on the sharing of latrines in 594 households (92 cases, 502 controls) in seven communities in Tanzania. Case households were those with at least one child with clinical signs of trachoma. The results showed that the latrine use was associated with a lower risk of trachoma, but there was no difference in the risk of developing trachoma between households that used community latrines versus private latrines (OR: 0.95; 95% CI: 0.55-1.67) [52].

Health Education 

The knowledge and attitudes of individuals and households in trachoma endemic countries play a significant role in their safety practices against trachoma. Mamo et al. conducted a study in the Tigray region of North Ethiopia that looked into assessing the knowledge, practices and attitudes of the local population with regard to trachoma. A study was conducted in two parts of the Tigray region. When assessing the knowledge of trachoma, the study found that awareness on routes of transmission was relatively low; only 54.6% of the participants knew that trachoma is a transmissible disease from person to person. A portion (35.6%) of the participants were aware that flies acted as a vector for the transmission of trachoma, and 24.7% knew that trachoma can be transmitted by the sharing of a contaminated towel. When assessing attitude, 95.4% agreed that the availability of an adequate water supply was an important factor for the prevention of trachoma, yet only 69.1% of the participants agreed that personal hygiene was an important factor for trachoma prevention [53].

When differentiating between those with good and poor knowledge of trachoma practices, the results showed that previously receiving health education with regard to trachoma had a significant independent association with good knowledge (95% CI: 1.91-8.79) and good attitudes (95% CI: 1.02-4.25). The study concluded that there is a large disparity in the knowledge and attitudes of the public in the Tigray region about trachoma and further efforts need to be made with respect to health education in order to correct their current malpractices, which are directly associated risk factors for trachoma [53].

The impact that health education programmes have on the prevalence of trachoma can be demonstrated in a study conducted by Edwards et al. [27]. The objective was to evaluate the impact of health education on the prevalence of trachoma in children aged three to nine years of age. The study was conducted in rural Ethiopia across 40 communities. The participants all came from randomly selected households and were within the age range of three to nine years. Several different methods were used to spread the knowledge of safety measures for trachoma, including radio broadcast, NGO activities based on SAFE and educational videos. The main outcome measure was the prevalence of active trachoma in children aged three to nine years and the knowledge of adults related to the nature and transmission of trachoma. Of the children that were followed up, 64% had active trachoma; this was an 8% reduction from the baseline level (95% CI: 4-12%; P < 0.001). A significant increase was found in the knowledge and awareness of trachoma. In both intervention groups, the odds of active trachoma were reduced (OR: 0.78; 95% CI: 0.53-1.16 and OR: 0.76; 0.95% CI: 0.48-1.21). Overall, this was a small yet statistically substantial decrease in the prevalence of active trachoma after the intervention of health education protocols [27].

Can Mass Antibiotic Treatment Alone Eliminate Trachoma?

In order to establish whether environmental intervention is required to eliminate trachoma, first, we must assess whether other strategies are enough, on their own, to eliminate trachoma. There have been extensive discussions whether solely mass antibiotic treatment can be sufficient in eliminating trachoma, thus making costs and efforts towards environmental improvement very limited, as it is not a priority. The WHO ‘SAFE’ strategy incorporates both medical and environmental interventions; nonetheless, several studies have investigated whether antibiotic treatment alone is sufficient. 

A study conducted by Chidambaram et al. looked at whether a single mass antibiotic distribution would be ample enough to eliminate trachoma after a 24-month follow-up. A longitudinal cohort study was conducted between 2003 and 2005 in the ‘Gurage zone’ of Ethiopia [54]. Eight villages were selected at random to assess for ocular *C. trachomatis* infection, and 15 untreated villages were selected at random one year to assess for a trend. Any resident in the eight selected villages that was aged 12 months or older were given a singular dosage of oral azithromycin. The main outcome measure of this piece of research was the incidence of ocular chlamydial infection in the intervention villages at two-, six-, 12-, 18- and 24-months' post mass antibiotic treatment in all children aged one to five years and also in untreated villages enrolled at 12 months [54].

In total, 515 participants were assessed for ocular chlamydial infection at the baseline level. Of the 515 participants, 87 were lost to follow-up as they did not attend follow-up examinations. Two months following the first treatment, the mean prevalence of the infection declined significantly from 43.5% (95% CI: 35.0-52.0%) to 5.1% (95% CI: 1.1-9.2%). After a 24-month follow-up, the prevalence of infection in seven out of eight villages increased after two months to an average of 11.3% [54]. Several other studies have also evidenced the use of a single antibiotic treatment to be insufficient to prevent infection from returning [55-57].

A study conducted in Ethiopia by Lakew et al. identified that the reoccurrence of infection after a single antibiotic treatment was common, and accordingly, a study was conducted to assess whether repeat scheduled treatments of azithromycin would work to further reduce the prevalence of infection and if it returns after the discontinuation of the distributions [57]. Children aged one to five years in 16 Ethiopian communities were randomly selected to monitor the rates of ocular chlamydial infection over four twice-yearly azithromycin distributions, which was over a two-year follow-up period after the last treatment. After the first mass azithromycin distribution at six months, the prevalence of infection reduced from 63.5% baseline to 11.5% (P < 0.0001). In the six months following the fourth and final distribution of azithromycin, the prevalence rate had dropped even further to 2.6% (P = 0.0004). Be that as it may, after the full follow-up period of 24 months, post final treatment, the prevalence rate significantly increased to 25.2% (P = 0.008). The overall trend in the 16 villages was that the mean prevalence decreased after each treatment yet returned slowly after treatments were discontinued, concluding that antibiotic distribution alone is not sufficient enough to eliminate trachoma [57]. 

The natural concerning question that leads on from this is that is if infection will just return into areas following the final treatment, can distribution of antibiotics ever be halted, and if so, when? A study conducted by Ray et al. looked to address this exact concern [58]. The research paper suggested ‘graduating communities when the prevalence of infection identified in children decreases below a threshold’, meaning that the treatment would be discontinued at this point. The testing could be done empirically, but the results would not be available for years. Therefore, a mathematical model was used for result predictions with separate graduation approaches in three different countries in Africa, namely, Tanzania, the Gambia and Ethiopia. Results showed the pre-treatment infection rates in children to be 16% in Tanzania, 9% in the Gambia and 64% in Ethiopia. At three years, the expected infection prevalence was gathered and assigned separate limits for graduation. The model forecasted that tri-annual treatments at 80% coverage would lower the *C. trachomatis* infection prevalence to 0.03% in Tanzania, 2.4% in the Gambia and 12.9% in Ethiopia. Should the graduation threshold be set at when infection drops lower than 5%, then the mean incidence of infection at three years was predicted to be 0.3%, 3.9% and 14.4% respectively. Graduations allowed for a reduction in the use of antibiotics in Tanzania by 63%, in the Gambia by 56% and in Ethiopia by 11% [58].

The study concluded that a policy that graduates communities when infection rates fall below 5% is reasonable and that treatment can be discontinued in the vast majority of communities, such as the Gambia and Tanzania, once this threshold has been reached. Having this threshold also allows for the more efficient use of antibiotic resources and could have a reduction in the amount of antibiotic used in distribution by greater than twofold [58].

Discussion

What Should Be the Focus: Medical or Environmental Intervention?

The use of mass antibiotic distribution has been at the core of the WHO SAFE strategy since the beginning of the GET 2020 campaign to treat ocular chlamydia infection. It has been debated at length whether the antibiotic use alone can eliminate the issue of trachoma in endemic countries. The evidence strongly suggests that antibiotic use is effective in lowering the prevalence of trachoma, with one single village study conducted by Solomon et al. showing that the use of azithromycin and tetracycline eye ointment reduced the prevalence of infection with *C. trachomatis* from 9.5% to 0.1% 24 months post treatment [59]. Evidence such as this would suggest that antibiotic use does maintain its effect long term, but other studies have shown rather different results, showing that the effect of antibiotic treatment weans after the completion of a treatment cycle.

Lakew et al. conducted a longer longitudinal assessment of *C. trachomatis* infection over 42 months following four semi-annual mass treatments across 16 different communities in the Gurage zone of Ethiopia [57]. This study showed that whilst the prevalence rates of trachoma significantly reduced by 52% in the first six months from 63.5% to 11.5%, after the discontinuation of treatment 24 months, post final treatment, the prevalence rates rose to 25.2%. Unlike the study conducted by Solomon et al., which was conducted in one single village, the study by Lakew et al. was operated across 16 villages with a longer follow-up period of 42 months, compared with 24 months. It is important to note that whilst the mean prevalence rates of trachoma were calculated across 16 villages, none of these villages performed exactly at the average mean level. This wide variation across these 16 villages strongly argues that studies conducted in lone villages, such as that by Solomon et al. amongst others, should be interpreted with a high degree of caution or even considered as unreliable.

It is important to note that in the study conducted by Lakew et al., none of the villages achieved total elimination of trachoma at any stage of the treatment cycle nor after the end of the 42 month follow-up. Re-infection occurred across all villages, despite the intense antibiotic distribution programme, and transmission was fast due to the fact that environmental improvement and behavioural habits, such as face washing and WASH measures, had not been strongly implemented. The message interpreted from this strongly suggests that the approach of eliminating trachoma solely on antibiotic distribution is highly unlikely to be successful. For this reason, the implementation of environmental improvement strategies backed by health education programmes must complement antibiotic distribution in order to achieve success.

In order for a country to be classified as trachoma-free, evidence is required to demonstrate that the elimination prevalence thresholds have been reached and maintained for at least a two-year period. In 2008, Ghana reached these prevalence thresholds. Since 2011, Ghana have been conducting their ‘trachoma surveillance strategy’, which are primarily based upon operating population-based surveys and community and school screening for clinical signs of trachoma. Between 2015 and 2016, a survey was organised to evaluate whether or not trachoma had indeed been eradicated as a public health crisis [60]. This survey was also able to highlight the most important factors that were key to Ghana’s elimination strategy. The study found that 17 out of 18 previously trachoma endemic districts maintained their elimination thresholds. Of the households in these districts, 75.9% (8,424) had access to an improved water source; this is an improvement of 27.3% from 2003. Moreover, in terms of facial cleanliness, only 5.1% of children were found to have ocular discharge [60]. Although mass antibiotic distribution was a fundamental element in Ghana’s success in eradicating trachoma, it is clear to see that this worked effectively alongside improved environmental factors, which contributed heavily to the maintenance of the elimination threshold trachoma prevalence levels. These same levels were not able to be achieved in programmes where antibiotic distribution was the sole interventional measure.

Overall, there is no room for doubt in the fact that mass distribution of antibiotics is the most efficient method of speedily reducing the rates of infection with *C. trachomatis*. Nevertheless, unless accompanied by effective facial cleanliness and environmental improvement strategies, trachoma will not be eradicated from the current affected areas.

What Is the More Effective Environmental Intervention in Lowering the Prevalence of Trachoma: Facial Cleanliness or Latrine Provision?

When analysing what the most effective environmental intervention is, in the battle eradicating trachoma, two crucial factors must be considered. The first being efficacy in reducing the prevalence rates of trachoma, and the second is practicality, with particular emphasis on cost effectiveness, as trachoma is a disease treated on a very limited budget. The study conducted by Abdou et al. analysed not only the importance of facial cleanliness on the prevalence on trachoma but also the provision of latrine facilities amongst other environmental factors [48]. This allowed for a direct comparison to be made.

Children with clean faces (i.e. no signs of uncleanliness, such as ocular/nasal discharge) but with flies on their faces had an increased likelihood of contracting trachoma in comparison with the children who had clean faces without flies on their face; however, no association was found between facial flies and infection. Given that it is a widely accepted fact that eye-seeking flies are a vector for *C. trachomatis*, the observation that facial flies were not associated with trachoma prevalence was unexpected. This study was in agreement with West et al., which highlighted that many studies may have incorrectly defined a ‘clean face’ by making the ‘absence of flies’ one of the requirements [32]. Conversely, despite the absence of flies not being linked with lower prevalence of trachoma, an unclean face was still found to be the most important personal characteristic associated with trachoma infection with a P value of <0.001. Children (with or without flies) with an unclean face had a three times greater likelihood of developing infection in comparison to those with clean faces. This conclusion is consistent with the available literature that highlights unclean faces as a strongly statistically significant risk factor for trachoma [61,62].

Abdou et al. also looked into whether latrine facilities were also associated with *C. trachomatis* infection. Only 39 children from the 635 children analysed had access to private latrine facilities, yet when the percentage of children with infection in the latrine and non-latrine groups were assessed and compared, the difference was innocuous. A proportion (20.5%) of children with access to latrine facilities were infected with *C. trachomatis;* this figure was only 0.8% greater (21.3%) in the group of children without latrine access, making the difference statistically insignificant. It is interesting to note that the study by Montgomery et al. also had similar findings when assessing the association between private latrine facilities and *C. trachomatis* infection. It was found that whilst latrine use was linked with a lower risk of trachoma, a significant distinction between the use of private and shared latrines on the risk of *C. trachomatis* infection was not found. Moreover, an increased quantity of households sharing a latrine did not have an impact on increased odds of trachoma [48].

Given that open defecation is still strongly associated with risk of trachoma [63], it should be encouraged that community latrines are still built in areas that are lacking, even basic levels of, latrine facilities. In the event that latrines are only located within a few households, those households with a latrine are to be encouraged to allow neighbours to use this facility. This is until there is either an accessible communal latrine is built or they have an in-built latrine in their own households. However, the findings from the available literature suggest that, in the rural areas of sub-Saharan Africa, efforts are best not spent on expensive latrine programmes that aim to provide facilities to every household. These efforts, rather, should be towards the sufficient facilitation of shared latrine use as it is more cost effective and there is no statistical difference between communal and private latrines on the risk of contracting trachoma as supported by Montgomery et al. [52] and Harding-Esch et al. [64] in the Gambia.

Facial cleanliness, therefore, would appear to be a greater cornerstone for trachoma prevention as opposed to latrine facilitation, both in terms of its association with risk of infection and cost effectiveness. Facial cleanliness incorporates two important factors: first, ocular and nasal discharge that can be transmitted from person to person is removed by face washing, and second, the removal of traces of food, mucous or other material decreases the attractiveness of eye-seeking flies, which act as a vector for *C. trachomatis*. Both of these roles, however, are dependent on water supply and the correct face washing tools. As found by Czerniewska et al., it is demonstrated that washing with soap had the greatest effect on removing visible signs of facial uncleanliness than washing with water alone [50]. 

Insufficient Water or Insufficient Knowledge?

A strong association was found between water access and a reduced risk of trachoma across several different studies [44-46]. Nevertheless, it is also imperative to note that water access does not automatically imply that the water is being used according to the correct prioritisation. It would not be a beyond-reasonable thought to suggest that the further a family is located from a source of water, the less water they are likely to have water in their household; thus, the children within that household are less likely to have clean faces. In spite of that, West et al. found that the distance to the nearest water source and the daily amount of water brought into the household were independent of each other and that children who lived further away from a water source had an increased chance of having unclean faces, irrespective of the quantity of water taken into the household. In fact, it was found that the amount daily water brought into the household was insignificant to the prevalence of trachoma, nor the chance of the children having unclean faces, as was also found by the study conducted by Bailey et al. [65].

These findings raise questions as to whether the challenge to eradicate trachoma lies in improving 'insufficient water supplies' or in educating the population in hyperendemic countries on how to make the maximum use of the water available. Oftentimes, insufficient water supply for face washing is determined by the number of children with unclean faces, but this should be interpreted with the context of utilisation priorities of the water available within the household. Health education, therefore, should be at the forefront of any trachoma elimination programme. Health education is likely to be cheaper than building new water supplies, and the intervention of health education programmes has been shown to have a statistically substantial impact on lowering the prevalence rates of trachoma within varying communities [53]. 

Trachoma and the Immune System

The current trachoma treatment measures are predominantly based around mass antibiotic distribution in hyperendemic areas. This approach is both logistically challenging and ineffective in the long term due to the shown re-infection rates post treatment [57]. The development for a vaccine would offer significant advantages, particularly by tackling the limitations of antibiotic treatment. These include antibiotic resistance (unlikely), personal antibiotic adherence failure, differing rates of mucosal absorption and heterotypic resistance in relation to populations of the organism [66]. Nonetheless, a limited understanding of both protective immunity and immunopathology of *C. trachomatis* remains a barrier for the development of a trachoma vaccine.

Studies conducted on human volunteers of ocular *C. trachomatis *offered indication that following infection, an immune response develops that is partially protective [67-69]. Conjunctival infection with *C. trachomatis* was proven to give rise to follicular/papillary conjunctivitis in the vast majority of volunteers after a period of two to 14 days. Volunteers that were previously infected were reinfected with the same serovar of *C. trachomatis*, and it was demonstrated that there was a reduced clinical response with reduced rates of re-isolation. The study detected that when an alternative serovar was utilized, the level of disease was similar to the primary infection, making the observed immunity serovar-specific. Trachoma models conducted on animal primates also observed that following primary infection, the secondary re-challenge presented with a less severe clinical presentation that resolved quicker [69].

The data suggest that the systems of protective immunity involve interferon-gamma (IFN-γ)-dependent cell-mediated immunity (CMI). Studies conducted on animals with genital chlamydial infection indicate that the solution of infection is determined principally via type-1 CD4+ T helper 1 (Th1) lymphocyte response that is principally facilitated via IFN-γ. Several different types of cells, such as Th1 lymphocytes, CD8+ cytotoxic T lymphocytes (CTLs) and natural killer (NK) cells, all produce IFN-γ [70]. The response of Th1 is characterised by the production of IFN-γ. It is believed that IFN-γ has several anti-chlamydial measures that are vital to the clearance of infection. The expression of Indoleamine-2,3-dioxygenase (IDO) is induced by IFN-γ, which in turn degrades tryptophan and thereby causes the levels intracellular tryptophan to decrease, which is a vital amino acid necessary for the metabolism of *C. trachomatis* [71].

The above outlined acquired T-cell-dependent CMI responses are believed to contribute to either protection against reinfection or a quicker resolution of re-infection. The issue still remains, however, that human evidence that resolution of ocular *C. trachomatis* infection is IFN-γ dependent is limited.

Silverstein in 1974 wrote a report about his findings with regard to the 'immunologic modulation of infectious disease pathogenesis', where he spoke about the immunopathogenesis of trachoma [72]. Silverstein suggested that the actual *C. trachomatis* only infects the conjunctival (and possibly corneal) epithelium of the eye and is never detected beneath the epithelial lining, making the infection rather innocuous. His main proposition, therefore, was that the tissue damage and scarring observed clinically is a result of a chronic immunopathogenic response, associated with Th1 and Th17. This proposal was supported by his findings that inflammation and consequent scarring were found at the stromal level of the conjunctiva, whereas the infection itself is located at the epithelium. Another key observational finding that supports Silverstein’s proposal has been supported by several other studies that show that observed conjunctival inflammation is commonly discovered where *C. trachomatis* is undetectable [73-75]. Longitudinal studies have also shown that episodes of active trachoma remain for a long time after the accompanying infectious outbreak has been cleared [75].

The expression of interleukin (IL)-17A, a pro-inflammatory cytokine (indicative of Th17 action), has been evidenced to be markedly raised in inflammatory trachoma. It is believed that IL-17A performs a function in fibrosis via the epithelial-mesenchymal transition and raised production of collagen [76]. A raised expression of matrix metalloproteinase MMP-9 (gelatinase-B) [77] is also found in scarring trachoma; this is a metalloproteinase that is not only induced by NK cells but also induced and regulated via the Th1 cytokine, IFN-γ. The expression of conjunctival MMP-9 has been found to be markedly raised in children with active trachoma [78].

Studies on genital infection with *C. trachomatis* show that the amount of IL-17 and IL-22 are far greater in infected women than non-infected women [79]. Mabey et al. noted that a crucial distinction to highlight between genital and ocular infections is the site at which the damaging sequence occurred. Ocularly, the damaging sequence appears at the location of the initial infection, the conjunctival epithelium, whereas in genital infection, the damaging sequelae occurs in the fallopian tubes as opposed to the cervix, the site of injection [80].

However, despite this, it is still important to recognise that there are many similarities between the pathology of *C. trachomatis* ocularly and genitally, involving an intricate interface of both innate and adaptive immune responses to chlamydia that has an influence on both pathology and protection. Any developed vaccine must certify that the immune responses produced by both the ocular and genital vaccines consist of the suitable groups of lymphocytes that will stimulate protection and lessen pathology in the individual [81].

Progress Towards the Trachoma Vaccine

In recent history, over the past 10 odd years, efforts towards creating a vaccine for *C. trachomatis* have been centred upon the utilization of recombinant proteins, used singly, or occasionally, as concoctions [82]. However, in spite of many attempts, the degree of 'protection' has remained limited. The template most generally employed to assess vaccines has been the *C. muridarum *mouse model [83], but a major challenge still remains as to how the data from mice will translate into human studies.

A study conducted by Kari et al. produced a live weakened (plasma-free) strain of *C. trachomatis* and assessed the efficacy on monkey models. The eyes of cynomolgus macaque monkeys were infected with a plasmid-deficient strain of *C. trachomatis*. The results from this study showed that the attenuated strain caused a short-lived infection that resolved spontaneously in comparison with the virulent wild-type strain, and most importantly, no ocular pathology was produced. The virulent wild-type strain was then used to challenge six monkeys that had received vaccinations and six monkeys that did not. Of the monkeys in the unvaccinated group, all six developed serious pathology and prolonged infections. Of the monkeys in the vaccinated group, three were fully protected from any ocular pathology, whereas the other three had partial protection and still had significant pathology [84].

These results are promising for future vaccine development, but the regulatory obligations concerned with the use of live attenuated vaccines necessitates the complete comprehension of the molecular processes underlying these plasma-free 'vaccine strains'. With reference to this, another recent breakthrough finding that can significantly aid in vaccine research is the capacity to manipulate chlamydia now genetically [82]. This outstanding accomplishment now gives vaccine research the potential to delete or inactivate key genes to aid with the understanding of their purpose in pathogenesis [84]. This ought to hopefully result in the production of a live attenuated trachoma vaccine that is incapable of causing adverse pathology. These ground-breaking developments gives optimism that the previously out of reach *C. trachomatis* vaccination may soon come into fruition. 

Limitations

As the GET 2020 project was only in the last few years, there was limited literature available evaluating the campaign and its success. The literature review searches were conducted on two scientific databases, OVID MEDLINE and OVID EMBASE, so there was a missed potential for finding literature on other scientific databases. Whilst the strict criterion applied to the searches aided in keeping the quantity of articles manageable, the number of papers selected was limited as a result. A significant limitation in the literature search conducted was the shortage of gold-standard randomised control trials concerning trachoma and environmental improvement. Consequently, as the studies did not meet the high-quality standard required, changing clinical practice becomes difficult as a result. Looking into the future, randomised control trials regarding the impact of environmental improvement strategies on trachoma needs to be conducted. The limited available literature also made comparing studies a difficult task. Different studies had different classification criterion for what defines environmental improvement, thus making direct comparisons and drawing conclusions challenging.

## Conclusions

When determining the best approach to eliminate trachoma, the most important factor to acknowledge is that a single-method approach is extremely unlikely to yield success. Although mass antibiotic treatment has been proven to have a great immediate impact on lowering the prevalence of trachoma than environmental improvement strategies, the literature shows that trachoma prevalence rates rise again following the completion of the treatment cycle showing that a single-method antibiotic approach alone cannot eliminate trachoma. An emphasis on long-term environmental methods for trachoma control is vital to the success of eliminating trachoma as a public health concern. 

Whilst the foundations of trachoma elimination will always remain, it is also important to acknowledge that the challenge the WHO faced with eliminating trachoma when the GET 2020 project was established in 1999 is vastly disparate to the challenge faced today. With trachoma being eliminated from various areas as of now, the challenge is now presented in smaller epidemiological situations, meaning that the intervention approach to elimination is required be tailored to specifically suit the local epidemiology at hand. These measures, however, are unsustainable without adequate health education promotion. With such limited costs and dry environmental conditions, water supply is a scarce resource that must be utilised efficiently. Early healthcare-seeking behaviour and the education of women who are often the primary caregivers of children must be actively pursued in the fight to eradicate trachoma.

## Appendices

Appendix A

**Table 1:** OVID Medline (1946-July 2021) Literature search conducted on OVID Medline

	Searches	Results
1	Exp trachoma/	278
2	(Ocular Chlamydia or Trachomatous Conjunctivitis or Chlamydia Trachomitis). mp. [mp=title, abstract, original title, name of substance word, subject heading word, floating sub-heading word, keyword heading word, organism supplementary concept word, protocol supplementary concept word, rare disease supplementary concept word, unique identifier, synonyms]	35
3	1 or 2	284
4	(Trachoma and Water) .mp. [mp=title, abstract, original title, name of substance word, subject heading word, floating sub-heading word, keyword heading word, organism supplementary concept word, protocol supplementary concept word, rare disease supplementary concept word, unique identifier, synonyms]	51
5	(Trachoma and Sanitation) .mp. [mp=title, abstract, original title, name of substance word, subject heading word, floating sub-heading word, keyword heading word, organism supplementary concept word, protocol supplementary concept word, rare disease supplementary concept word, unique identifier, synonyms]	43
6	(Trachoma and Hygiene) mp. [mp=title, abstract, original title, name of substance word, subject heading word, floating sub-heading word, keyword heading word, organism supplementary concept word, protocol supplementary concept word, rare disease supplementary concept word, unique identifier, synonyms]	41
7	(Trachoma and GET 2020) mp. [mp=title, abstract, original title, name of substance word, subject heading word, floating sub-heading word, keyword heading word, organism supplementary concept word, protocol supplementary concept word, rare disease supplementary concept word, unique identifier, synonyms]	2
8	(Trachoma and Socio-economic) mp. [mp=title, abstract, original title, name of substance word, subject heading word, floating sub-heading word, keyword heading word, organism supplementary concept word, protocol supplementary concept word, rare disease supplementary concept word, unique identifier, synonyms]	2
9	4 or 5 or 6 or 7 or 8	75
10	3 and 9	64

Appendix B

**Table 2:** OVID Embase (1946-July 2021) Literature search Conducted on OVID Embase

	Searches	Results
1	Exp trachoma/	3513
2	(Ocular Chlamydia or Trachomatous Conjunctivitis or Chlamydia Trachomitis). mp. [mp=title, abstract, heading word, drug trade name, original title, device manufacturer, drug manufacturer, device trade name, keyword, floating subheading word, candidate term word]	172
3	1 or 2	3550
4	(Trachoma and Water) mp. [mp=title, abstract, heading word, drug trade name, original title, device manufacturer, drug manufacturer, device trade name, keyword, floating subheading word, candidate term word]	300
5	(Trachoma and Sanitation) mp. [mp=title, abstract, heading word, drug trade name, original title, device manufacturer, drug manufacturer, device trade name, keyword, floating subheading word, candidate term word]	281
6	(Trachoma and Hygiene) mp. [mp=title, abstract, heading word, drug trade name, original title, device manufacturer, drug manufacturer, device trade name, keyword, floating subheading word, candidate term word]	460
7	(Trachoma and GET 2020) mp. [mp=title, abstract, heading word, drug trade name, original title, device manufacturer, drug manufacturer, device trade name, keyword, floating subheading word, candidate term word]	24
8	(Trachoma and Socio-economic) mp. [mp=title, abstract, heading word, drug trade name, original title, device manufacturer, drug manufacturer, device trade name, keyword, floating subheading word, candidate term word]	36
9	4 or 5 or 6 or 7 or 8	684
10	3 and 9	649
